# Gastric adenocarcinoma incidence in the province of Messina (Insular Italy): A cancer registry study

**DOI:** 10.3892/ol.2013.1758

**Published:** 2013-12-12

**Authors:** ROSARIO A. CARUSO, ELEONORA IRATO, GIOVANNI BRANCA, GIUSEPPE FINOCCHIARO, FRANCESCO FEDELE, ALESSIA ARNESE

**Affiliations:** 1 Department of Human Pathology, University Hospital, Messina I-98125, Italy; 2 Social Health District, Taranto I-74100, Italy

**Keywords:** gastric adenocarcinoma, cancer registry study, Messina province

## Abstract

The incidence of cancer by age, gender and tumor type at a population-based level is infrequently investigated. The aim of the present study was to describe the burden and outcome of gastric carcinomas (excluding cancers of the esophagogastric junction) experienced by the elderly, particularly for patients aged ≥81 years. A population-based series of 322 patients exhibiting gastric cancer, diagnosed between 2003 and 2005 and from the province of Messina (insular Italy; population, 662,450) was used. The median age of patients at the time of diagnosis was 72 years. The patients were categorized into three age groups according to interquartile range values, <64, 65–80 and >81 years. The cancer-specific survival rate at five years was lowest in the very elderly (P<0.001). Patients aged ≥81 years were less likely to receive surgery than younger patients (44 vs. 55 vs. 22% for the <64, 65–80 and >81 years age groups, respectively; P<0.01). In the resected cases, very elderly patients (age, >81 years) were more likely than younger patients to exhibit advanced stage pathological tumor-node-metastasis (P<0.05). It was concluded that patients aged ≥81 years accounted for 25% of total gastric carcinomas, were less likely to receive surgery and experienced worse outcomes when compared with younger patients.

## Introduction

Cancer is a disease of aging. The elderly (individuals aged ≥65 years) account for 61% of novel cancer cases and 70% of cancer-related fatalities, and exhibit ~11 times the cancer risk compared with people aged <65 years (1). Italy ranks as the oldest population in the world; in the year 2000, 18% of the Italian population was ≥65 years and by 2030, 27% of Italy’s population will be aged ≥65 years (2).

The influence of patient age and gender, and the location of the carcinoma within the stomach at diagnosis, have been central to understanding the etiology of gastric carcinomas (3). Age-standardized and cumulative incidence rates of gastric cancer in males are approximately double the rates in females (4). However, the gender ratio of the incidence rates varies with age, forming a low-high-low pattern, which appears to be unique to stomach cancer (4). Within Europe, Italy maintains one of the highest fatality rates for stomach neoplasms, with marked internal variation (5,6). The data provided by several cancer registries located across the country confirmed that the incidence of gastric cancer and mortality rates vary widely, indicating that the highest risk is observed in central-northern regions and the lowest risk in the south of Italy (6–8).

A retrospective population-based study was conducted over three years (2003–2005), with an extensive search for all cases of gastric cancer. The data was analyzed to assess the burden and outcome of gastric carcinomas (excluding cancers of the esophagogastric junction) in the elderly, particularly for patients aged ≥81 years.

## Patients and methods

### Data source

A population-based cancer registry was compiled in 2005 to cover the resident population of the province of Messina, Italy (662,450 residents according to the 2001 census). The cancer registry of Messina is part of the Integrated Cancer Registry of eastern Sicily, which includes Siracusa, Enna and Catania and has collated data of patients that were diagnosed with cancer between January 2003 and December 2005. The registry is defined by the National Committee of Registries (Milan, Italy) and operates in compliance with the recommendations of the International Agency for Research on Cancer (9).

The incidence of primary cancers of the stomach were analyzed, excluding cases of lymphoma and leukemia (histology codes, 9590–9989), mesothelioma (histology codes, 9050–9055 ) and Kaposi sarcoma (histology code, 9140) (10). Therefore, patients for whom gastric adenocarcinoma was their first and only cancer diagnosis were included.

The clinical and pathological parameters that were analyzed for the patients were gender, age, carcinoma site and histological type. In addition, depth of invasion into the gastric wall and the nodal status (the number of regional lymph nodes examined and the number of those invaded) were analyzed in the resected cases. The tumors were restaged according to the tumor-node metastasis (TNM) classification system (11).

Notes obtained from surgical procedures and the results of pathological tests were the preferred information sources for tumor site identification; if these sources were unavailable, endoscopic or radiologic reports were evaluated. In addition, physician documentation and descriptions of patients at the time of diagnosis were used to determine tumor location. With regard to tumor location within the stomach, cases were grouped as follows: Proximal (fundus C16.1), distal (body C16.2, lesser curvature C16.5, antrum C16.3 and pylorus C16.4, greater curvature C16.6) and overlapping/unknown (overlapping sites or unknown primary site; C16.8). Patients were excluded if they exhibited a World Health Organization (WHO)-defined gastroesophageal junction tumor (a tumor that spanned the esophageal junction, regardless of the location of the majority of the tumor) (12).

The histological classification of gastric carcinomas, into intestinal or diffuse type, was based on the criteria proposed by Lauren (13): Diffuse types included signet ring cell carcinomas (WHO histological classification no. 8490), diffuse carcinoma (no. 8145) and linitis plastica (no. 8142); intestinal types included carcinoma (not otherwise specified; no. 8010), adenocarcinoma (not otherwise specified; no. 8140), tubular (no. 8211) and intestinal type (no. 8144).

### Statistical analysis

The categorical data were summarized using frequencies and percentages. The distribution of the continuous variables was assessed; however, age at diagnosis was not normally distributed. Therefore, these data were summarized using medians and interquartile ranges and, according to the values of the interquartile range, the patients were categorized into three age groups; <64, 65–80 and >81 years. The predominant clinicopathological results were compared between age groups. Group comparisons of categorical variables were performed using the χ^2^ test or, for small sample sizes, Fisher’s exact test.

The Kaplan-Meier method (14) was used to estimate cancer-specific survival rates. According to Sarfati *et al* (15), fatalities not resulting from a patient’s underlying cancer were treated as losses to follow-up at the date of mortality, assuming that the cancer fatalities were independent of those resulting from alternative causes. Survival times were measured in months and were censored at the date of mortality from causes other than the underlying cancer, or on December 31st, 2011 (whichever occurred first). The survival curves did not start at 100% as certain patients succumbed to their disease within a month of diagnosis, resulting in a zero survival time as months were used for the calculations. The survival curves were compared using the log-rank test and the statistical analysis was performed using Stata/MP software (Stata Corp., College Station, TX, USA). P<0.05 was considered to indicate a statistically significant difference.

## Results

**Figure 1:**
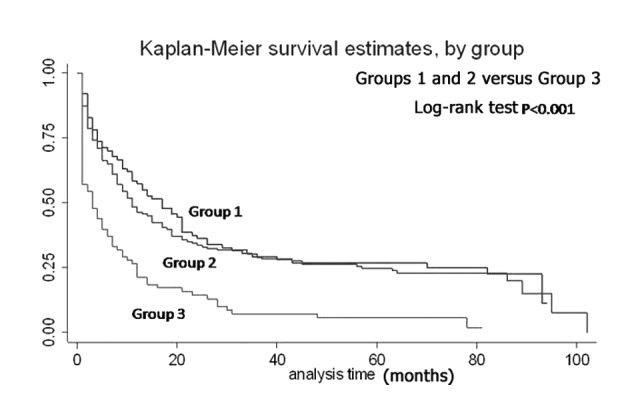
Kaplan-Meier survival curve demonstrating cancer-specific survival, by age, for 322 gastic carcinoma patients. Group 1, patients aged <64 years; Group 2, patients aged 65–80 years; Group 3, patients aged >81 years. P<0.001, groups 1 and 2 vs. group 3.

Between 2003 and 2005, 322 novel cases of gastric carcinoma were identified from the cancer registry of the province of Messina, which corresponded to a mean annual age-standardized incidence rate of 18.52 per 100,000 in males and 13.96 per 100,000 in females (age standardized to the world standard population). The mean patient age at diagnosis was 70 years, whereas the median age was 72 years (the 25th–75th percentile range was 64–80 years; thus, according to the values of the 25th–75th percentile, the patients were categorized into three age groups; <64, 65–80 and >81 years). There was a greater proportion of males in the three age groups. The male-to-female ratio was 1.12 in the younger age group (<64 years) but increased to a value of 1.83 in older patients (65–80 years), which subsequently decreased and attained levels marginally higher (1.19) in patients aged ≥81 years. Overlapping/unknown primary tumor location amounted to 39.44% and was significantly associated with advanced age (i.e. patients aged 65–80 years, P<0.05; patients ≥81 years, P<0.01; Table I). At the time of analysis, 58 patients had survived for more than five years, 260 had succumbed to gastric carcinoma or tumor metastasis within five years and four had succumbed to other diseases within five years. Cancer-specific survival rate at five years was significantly lower in the very elderly patients (P<0.001; Fig. 1). Among the 322 gastric carcinoma cases analyzed in the present study, 147 (45.65%) underwent a total or subtotal gastrectomy with curative intent, 48 (14.9%) received a laparatomy or palliative surgery and 127 (39.45%) did not receive any surgical treatment. Certain patients did not undergo resection of the tumor due to the presence of unresectable disease as a result of metastasis; these included liver metastasis, peritoneal dissemination and distant lymph node involvement. Other patients did not undergo resection due to the presence of severe complications, such as chronic heart failure, myocardial infarction, diabetes and aspiration pneumonia.

In the resected cases, very elderly patients were more likely than younger patients to exhibit an advanced pathological TNM stage (P<0.05). In addition, very elderly patients were more likely to present with higher proportions of T3, T4, N2, N3 and M1 disease stages when compared with the two younger age groups (P<0.05; Table II). Early gastric cancer (carcinomas confined to the gastric mucosa and submucosa) amounted to 14 (9.5%) out of 147 resected gastric carcinomas. No statistically significant correlations were identified between the three age groups and the proportion of patients who had ≥15 lymph nodes examined following gastric surgery, gender and histological type.

## Discussion

The incidence of stomach cancer and mortality in the north and center of Italy are estimated to be higher than those in the south and insular Italy, for males and females (8). In 2005, the incidence rates for males and females were 22 and 10 per 100,000, respectively, in the north; 24 and 11 per 100,000 in the center, respectively, and 18 and nine per 100,000 in the south, respectively, (8). Between 2003 and 2005, the age-standardized average annual incidence rates of stomach cancer for males and females in the province of Messina were 18.52 and 13.96 per 100,000, respectively. The age-standardized average annual incidence rate of stomach cancer for males, in the province of Messina, was analogous for males and higher for females with respect to that observed in the south and insular Italy (8).

In the present study, the patients were categorized by age, <64, 65–80 and >80 years, and the rationale was two-fold; the categories reflected the non-normally distributed age of the patients (median age, 72 years and interquartile range, 64–80 years) and followed biological logic. The majority of predominant types of cancer are diagnosed in people aged ≥65 years; thus, more than half (56%) of recently diagnosed cancer patients and 71% of cancer fatalities are in this age group (2,16).

In the unselected series that was analyzed in the present study, gastric carcinoma was a disease of elderly patients (25th percentile; age, 64 years) and was predominantly detected in the advanced stages. The cancer-specific survival rate at five years was lower in the very elderly (P<0.001). The data indicated that overlapping/unknown primary tumor location progressively increased with age and occurred significantly more often in middle-aged (P<0.05) and elderly patients (P<0.01). Moreover, patients aged ≥81 years were less likely to receive surgical therapy than younger patients (P<0.01). One possible explanation is the delay in diagnosis that may enable gastric carcinoma cases in the older age group (>81 years) to reach an advanced stage prior to the implementation of definitive diagnostic tests (including surgery) (16,17). Elderly patients may consider certain cancer symptoms to be a normal aspect of the ageing process, or relate their symptoms to common illnesses and, therefore, do not correctly interpret the symptoms as early-warning signals. Moreover, problems experienced by the elderly, such as social isolation due to the loss of a partner or friends, the distance to relatives, limited mobility, hearing or visual loss, other physical handicaps or previously existing diseases, may preoccupy the patient rather than the cancerous disease, which the patient may not be accustomed to discussing openly. Thus, a developing malignant disease may be neglected, as the symptoms do not influence daily routines (16,17).

The present study used cancer-specific survival rather than relative survival, which is considered to be the predominant measure used in population-based cancer survival studies (18). In population-based cancer registries, cause of mortality is obtained from death certificates, which are often inaccurate. Relative survival is calculated using life tables and is defined as the ratio of observed all-cause survival to expected survival, as obtained from The National Institute for Statistics life tables for selected periods. However, although this approach obviates the requirement to categorize individual fatalities, it introduces the significance of an external comparison group and a resulting assumption that the comparisons are valid (15). Recently, Sarfati *et al* (15) suggested that cause-specific survival and relative survival are potentially valid epidemiological methods in population-based cancer survival studies. Furthermore, the selection of the method is dependent upon the study objectives, the type of data available and the appropriateness of the assumptions underlying the two methods; particularly the availability of accurate cause of mortality data for cancer specific analysis. In the present study, the underlying cause of mortality was accurately defined as the disease or injury, which initiated the series of morbid events leading directly to mortality (19,20). Moreover, the cancer registry from the present study, was based on death certificates, accessed data on the specific cause of mortality and the type of cancer treatment.

There were several predominant limitations of the present study. It was a retrospective study and the majority of surgical procedures were performed by different surgeons using non-standardized surgical procedures. This resulted in inconsistent lymphadenectomy and collection of resection specimens; the extent of nodal involvement may, therefore, be underestimated. One limitation of the dataset was the relatively large proportion of patients with an overlapping/unknown anatomical subsite (39.44%); thus, certain patients were not able to be assigned to either the cardia or the non-cardia subgroup. However, in the present study, the gastric carcinomas were restaged according to the seventh edition of the TNM classification system (11), which is a crucial tool for treatment planning in oncology and for assessing the prognosis of a patient. The seventh edition of the TNM staging system for gastric cancer appeared to provide an improved categorized grouping than the sixth edition of the TNM, particularly between T2 and T3, and N1 and N2 tumors. The seventh edition of the TNM classification system hallmarks substantial changes for gastric cancer, which may influence treatment and provide the basis of future clinical studies.

In conclusion, the present study generated significant information concerning the burden of gastric carcinoma in the province of Messina and the outcomes experienced by very elderly patients (≥81 years). Thus, addressing the potential barriers to the optimal care of very elderly patients with prospective gastric cancer is a requirement, thus enabling effective treatments to be tailored to this group of patients.

## Figures and Tables

**Table I:** Demographic and clinical features of patients exhibiting gastric adenocarcinomas, stratified by age at the time of diagnosis (2003–2005).

	No. patients (%)			P-value		
		
Variables	<64 years (n=87)	65–80 years (n=156)	>81 years (n=79)	(<64 vs. >81 years)	(<64 vs. 65–80 years)	(65–80 vs. >81 years)
Gender				0.070	0.841	0.125
Male	46 (52.9)	101 (64.7)	43 (54.4)			
Female	41 (47.1)	55 (35.3)	36 (45.6)			
Primary tumor location				0.145	<0.05	<0.01
Proximal	2 (2.3)	13 (8.3)	3 (3.8)			
Distal	52 (59.8)	93 (59.6)	32 (40.5)			
Overlapping/unknown	33 (37.9)	50 (32.1)	44 (55.7)			
Undergoing curative surgery				0.123	<0.05	<0.01
Yes	39 (44.8)	86 (55.1)	22 (27.8)			
No	48 (55.2)	70 (44.9)	57 (2.2)			

**Table II:** Clinicopathological features of patients who underwent cancer-directed surgery, stratified by age at the time of diagnosis (2003–2005).

	No. patients (%)			P-value		
		
Variables	<64 years (n=39)	65–80 years (n=86)	>81 years (n=22)	(<64 vs. 65–80 years)	(<64 vs. >81 years)	(65–80 vs. >81 years)
No. of lymph nodes examined				0.607	0.871	0.816
<15	15 (38.50)	29 (33.70)	8 (36.30)			
>15	24 (61.50)	57 (66.30)	14 (63.70)			
pTNM stage				0.433	0.207	<0.05
IA	1 (2.60)	9 (10.50)	0 (0.00)			
IB	4 (10.20)	10 (11.60)	4 (18.20)			
IIA	5 (12.80)	14 (16.30)	0 (0.00)			
IIB	7 (17.90)	11 (12.80)	2 (9.10)			
IIIA	9 (23.10)	13 (15.10)	1 (4.50)			
IIIB	8 (20.50)	13 (15.10)	6 (27.30)			
IIIC	1 (2.60)	1 (1.20)	0 (0.00)			
IV	4 (10.30)	15 (17.40)	9 (40.90)			
T-stage				0.797	0.201	<0.05
T1	2 (5.12)	11 (12.79)	0 (0.00)			
T2	16 (41.02)	36 (41.86)	5 (22.72)			
T3	17 (43.58)	33 (38.37)	5 (22.72)			
T4	4 (10.25)	6 (6.97)	12 (54.54)			
N-stage				0.119	0.456	<0.05
N0	6 (15.40)	30 (34.88)	1 (4.50)			
N1	10 (25.60)	16 (18.60)	5 (22.70)			
N2	10 (25.60)	22 (25.58)	8 (36.40)			
N3	13 (33.40)	18 (20.93)	8 (36.40)			
M-stage				0.300	<0.01	<0.05
M0	35 (89.70)	71 (82.60)	13 (59.00)			
M1	4 (10.30)	15 (17.40)	9 (41.00)			
Cancer stage				0.342	1.000	0.272
Early gastric cancer	2 (5.10)	11 (12.80)	1 (4.50)			
Advanced gastric cancer	37 (94.90)	75 (87.20)	21 (95.50)			
Histological type				0.130	0.113	0.114
Intestinal	10 (25.60)	34 (39.50)	10 (45.50)			
Diffuse	29 (74.40)	52 (60.50)	12 (54.50)			

pTNM, pathological tumor-node-metastasis.
